# Medical Opinion and Movement

**Published:** 1907-01-12

**Authors:** 


### Medical Opinion and Movement.

The cry of "overcrowding" is fairly general, and applies to most professions and trades. We are not aware, however, that it has made the same impression, as shown by a reduction in the entries to other professions, as it appears to have done in the case of medicine. In many countries this falling-off in the number of medical students in recent years is very marked. In the United States in 1904 there were 28,142 medical students, whereas in 1905 there were only 26,137. The homœopathic schools show the most marked reductions. In 1900 they had 1,509 students, as against 1,104 in 1905. In Germany the number of students decreased from 8,513 in 1888 to 6,232 in 1903, and in France between 1895 and 1905 the numbers have fallen from 7,779 to 6,763. In this country, also, the idea is gaining ground that an average income of £300 a year is hardly sufficient compensation for a strenuous life preceded by an expensive and arduous education.

The ability of sodium citrate to render the casein of cow's milk more easy of digestion for infants, as originally suggested by Wright, is receiving general confirmation from the profession. It will be remembered that Wright's theory was based upon laboratory experiments, from which he concluded that, by precipitating some of the calcium salts in the milk with sodium citrate, the casein of cow's milk could be made to form clots in the stomach more resembling those of human milk. In France and America, as well as here, several physicians have recently published reports showing the satisfactory results obtained by administration of sodium citrate in those cases in which the infants show an inability to digest the casein of cow's milk in the ordinary way. The usual dose is one to three grains per ounce of milk, and should be prescribed in aqueous solution with a few drops of spirit of chloroform to prevent the formation of moulds. It is best made up so that one drachm of the solution added to each feed will give the necessary quantity of the sodium salt.

It is probably not very widely known in this country that the Dominion of Canada possesses no general register with a recognised standard of qualification for the medical profession. Each province possesses the right to establish its own licensing authority, and medical men, qualified and registered in one province, are not allowed to practise in an adjoining province under penalty of a fine. The conditions for registration of general practitioners who have qualified elsewhere than within the province vary considerably for the different provinces. The preliminary examinations are generally remitted, and in some cases the finals also; but the full fees always have to be paid. Ontario, which is reputed to have the highest standard of medical education, also makes the most stringent demands upon those presenting themselves for registration within the province. Attempts have been made to arrange for a general registration of medical men for the Dominion, but so far Quebec has objected on the ground that, owing to a large French population, there would be no advantage to the province by such a measure. If a greater reciprocity is to be looked for between the Dominion and the Mother Country, as was foreshadowed recently by the President of the General Medical Council, a general registration for the colony would appear to be a necessary preliminary.

The expectations aroused by the discovery of the tubercle bacillus by Koch and the diphtheritic antitoxin by Behring have not been fulfilled. The arduous labours of many brilliant workers in the field of bacteriology have done much to elucidate the processes of disease and to help us in our methods of prophylaxis and treatment; but we are still without a specific for tuberculosis, and we are still in the dark as to the cause of cancer or the means to combat it. It is not unreasonable, then, that a return should be made to lines of research which have already yielded results quite comparable in efficacy to those of serum therapy. In a recent communication to the Société de Médicine de Paris, Dr. L. Lematte has made out a well-reasoned case for a more thorough investigation into the therapeutic possibilities of the metals as specific bactericides. In mercury we have a definite specific against the ravages of the syphilitic microbe, and there is no *à priori* reason why other metals or combinations of metals should not have a like specific action on the morbid processes of the tubercle bacillus or other pathogenic micro-organisms.

For some years the chemist and bio-chemist have been so absorbed in the analyses and syntheses of complex organic compounds that the *rôle* played by the inorganic elements in the animal metabolism has been largely overlooked. The recent discoveries in chemical physics, notably the advances in the knowledge of the nature of chemical action and the interaction of the anions and kations, have again attracted the attention of investigators to the importance of the metallic ions in biological processes, and evidence is accumulating to show that these metallic ions are an essential factor in the oxidations, reductions, and other metabolic processes of living organisms. The necessary presence of calcium in the processes of blood coagulation and the clotting of milk is already well known. Professor Loeb has based a theory of irritability of tissue and muscular contraction upon the interaction of certain basic ions, notably sodium and calcium, and he has shown that eggs of a certain sea-urchin will develop, by appropriate treatment with sea-water slightly altered in constitution, without fertilisation by spermatozoa. These and similar facts indicate clearly the importance of minerals in the life processes. Dr. Lematte claims to have obtained favourable results by interstitial injection of chloride of gold in cases of inoperable cancer, but he is not yet prepared to say that he has found in gold a specific for cancer comparable to mercury in syphilis.

